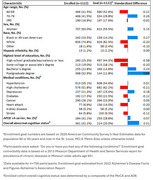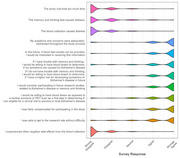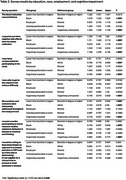# Participant Survey Results in a Diverse Community‐Based Alzheimer’s Disease Blood Test Study

**DOI:** 10.1002/alz70861_108690

**Published:** 2025-12-23

**Authors:** Melanie Burton, Melody Li, Lisa Soke, WeiChen Chang, Randall J. Bateman

**Affiliations:** ^1^ Washington University School of Medicine, Saint Louis, MO USA; ^2^ The Tracy Family SILQ Center, St. Louis, MO USA; ^3^ Knight Alzheimer Disease Research Center, Washington University School of Medicine, St. Louis, MO USA

## Abstract

**Background:**

A key component of Alzheimer's disease (AD) blood tests’ efficacy in real‐world clinical populations is ensuring its practicality and generalizability in diverse demographic groups. The Study to Evaluate Amyloid in Blood and Imaging Related to Dementia (SEABIRD) enrolled 1,122 participants to determine acceptability and validity (relative to amyloid PET) of blood plasma tests in a community‐based sample of older adults, and considered the impact of factors such as age, race, education, cognition, *APOE* genotype, and medical conditions on outcomes of the blood test.

**Method:**

SEABIRD measured plasma amyloid‐β 42/40 and phospho‐tau 217 in a diverse population of older adults (aged 60+) in the Saint Louis, Missouri, USA area. Participants completed blood collection, cognitive screening (AD8^®^ dementia screening interview and Montreal Cognitive Assessment [MoCA]), and a survey about their experience and perceptions.

**Result:**

23.5% of 1,122 participants identified as Black or African American (AA) (Table 1) and 85.2% reported having one or more of the following medical conditions: hypertension, high cholesterol, depression, diabetes, cancer, heart attack, kidney disease, stroke. Overall, and across demographic groups, participants reported positive study experiences. Approximately 92% disagreed that the blood collection caused distress. Nearly 84% agreed they would prefer blood draws over procedures such as lumbar punctures or PET scans (Figure 1). Participants who were Black or AA, had less than a bachelor’s degree, and were cognitively impaired were significantly less likely to be satisfied with compensation and ease of participation, and to express willingness to participate in future AD studies compared to white and highly educated individuals (Table 2).

**Conclusion:**

An AD blood test can be a feasible and widely used screening tool in a diverse population. To continue improving AD research, it is imperative to consider historically underrepresented populations and the barriers that may affect their initial and continuing participation. Broadening recruitment efforts to include varied sources across many populations, proactively addressing transportation needs, tailoring experiences to be inclusive of education levels and physical abilities, and careful consideration of compensation are some aspects of clinical studies that can create a positive impact on inclusivity and provide real‐world application to AD biomarker testing.